# A shift in psychiatry through AI? Ethical challenges

**DOI:** 10.1186/s12991-023-00476-9

**Published:** 2023-11-02

**Authors:** Saskia Wilhelmy, Giancarlo Giupponi, Dominik Groß, Klaus Eisendle, Andreas Conca

**Affiliations:** 1 Institute for History, Theory and Ethics in Medicine, University Hospital, RWTH Aachen University, Wendlingweg 2, 5074 Aachen, Germany; 2 Academic Teaching Department of Psychiatry, Central Hospital, Sanitary Agency of South Tyrol, Via Lorenz Böhler 5, 39100 Bolzano, Italy; 3 Institute of General Practice and Public Health, Provincial College for Health Professions Claudiana, Lorenz-Böhler-Straße 13, 39100 Bolzano, Italy

**Keywords:** Artificial intelligence, Digital transformation, Psychiatry, Bioethics, Patient–physician relationship

## Abstract

The digital transformation has made its way into many areas of society, including medicine. While AI-based systems are widespread in medical disciplines, their use in psychiatry is progressing more slowly. However, they promise to revolutionize psychiatric practice in terms of prevention options, diagnostics, or even therapy. Psychiatry is in the midst of this digital transformation, so the question is no longer “whether” to use technology, but “how” we can use it to achieve goals of progress or improvement. The aim of this article is to argue that this revolution brings not only new opportunities but also new ethical challenges for psychiatry, especially with regard to safety, responsibility, autonomy, or transparency. As an example, the relationship between doctor and patient in psychiatry will be addressed, in which digitization is also leading to ethically relevant changes. Ethical reflection on the use of AI systems offers the opportunity to accompany these changes carefully in order to take advantage of the benefits that this change brings. The focus should therefore always be on balancing what is technically possible with what is ethically necessary.

## Background

Digital transformation has taken hold in many areas of society. Increasingly, sophisticated technical innovations enable the continuous use and adaptation of information and communication technology. This technological revolution is omnipresent and generates discourses at the level of society as a whole. Transformation is also evident in medicine, where the era of “medicine 4.0” has already been ushered in, promising greater efficiency in individual patient care, the healthcare system, and medical research [1]. Buzzwords such as “digitization”, “big data” and “artificial intelligence (AI)” point the way to the future of digital health. While AI-based systems are widely used in disciplines such as radiology [2] or ophthalmology [3], their use in psychiatry, as a “talking” medical discipline, amounts to a Copernican revolution [4]. This revolution brings both new opportunities and ethical challenges.

## Main text

There are reasons why digital transformation is tantamount to a “slow” revolution for psychiatry. Brunn et al. were able to identify challenges that have an influence on the integration of AI applications: skeptical attitudes of psychiatrists toward AI, potential obsolescence of psychiatrists, and potential loss of definitional authority through AI [5]. User acceptance has a pivotal impact on the implementation of AI. Furthermore, technologies reflect and influence social structures—e.g. they shape communication and interpersonal relationships [6]. At the same time, they can give rise to developments that in turn can become drivers of insecurities and pathologies [7]. This continues to raise questions about the interdependence of technologies and society, thus also for psychiatry and which changes it will be subject to [5]. A survey of psychiatrists on the impact of AI and machine learning (ML) addressed this question. The study found that one in two psychiatrists predicted that their professional field will change significantly in the future.The majority of respondents do not believe that AI/ML could or will ever replace their work as psychiatrists, but that time-consuming work (e.g. documenting) will be transferred to AI/ML systems [8].

In addition to the physical, psychiatry focuses on the psyche and the brain of humans. In diagnostics and therapy, it thus faces the challenge of identifying and taking into account factors that ultimately influence the human psyche and brain. This is a challenge that has not been met primarily by technology. Nonetheless, or precisely because psychiatry is directly intertwined with the social matrix, AI-powered technology has found its way into it. In particular, this has been spurred by phenomena that psychiatry has faced in recent years, leading to calls for supportive or transformative technologies; e.g. regarding the COVID-19 pandemic, natural disasters, or war conflicts [9]. Digitalization has a stake in the crisis-ridden social matrix and at the same time embodies the tool as part of the coping process. This has led to increased engagement in research and clinical implementation of innovative technologies, which come with challenges, e.g. regarding research ethics standards such as transparency or reproducibility of information [10].

The emergence of technological innovations has thus triggered a dynamic in medicine in which the assessment of the use of such systems constantly oscillates between opposites: opportunities and hope on the one hand, risks and skepticism on the other [11]. A conclusive evaluation, e.g. with regard to possible risks or benefits, is often not possible, but rather a constant evaluation of the technology used is required. This is essential due to the rapid technological progress, which has also led to an acceleration and complexity of knowledge in medicine: today, medicine has a half-life of about 1–2 years, in the future it will certainly be even shorter [12].

### Ethical considerations on AI

Technological upheavals, such as the introduction of AI in society, simultaneously generate ethical challenges, which the European Union addressed in 2019 by introducing general ethical guidelines for the development, deployment and use of AI [13]: “Its central concern is to identify how AI can advance or raise concerns to the good life of individuals, whether in terms of quality of life, or human autonomy and freedom necessary for a democratic society” [13]. These guidelines concern the society as a whole, which is why they are formulated in an open manner, with the indication that they can be adapted and evaluated depending on the scope of application of AI. In addition to fundamental rights (such as respect for human dignity), the guidelines specify four non-hierarchical ethical principles that should be considered: respect for human autonomy, prevention of harm, fairness, and explicability [13]. These principles, which serve to protect humans interacting with AI, reflect ethical values that are also relevant in medicine when dealing with patients and can be found in the “principlism” established by Beauchamp and Childress. Their principles include (1) respect for human autonomy, (2) nonmaleficence, (3) beneficence, and (4) justice [14]. Unlike Beauchamp and Childress, the European Commission uses the principles mentioned to be taken into account as fixed values and not to be weighed against each other. When considering and evaluating AI applications in psychiatry, it makes sense to consult not only general but also medical-specific ethical guidelines—especially when AI is used with vulnerable groups. To this end, various ethical criteria in the use of technology in medicine provide guidance for conducting an ethical evaluation, e.g. with regard to self-determination, safety, privacy, or fairness [15, 16].

### Ethical challenges in psychiatry: doctor–patient interaction and AI

But what ethical challenges arise from the use of AI in psychiatry? This question aims at the ethical acceptability of using AI systems in this context. Various stakeholders (such as patients, relatives, or medical, nursing, and technical staff) involved in psychiatry and in the digitization process play a role. To illustrate the changes in the interpersonal interaction of these stakeholders, the doctor–patient relationship is considered as an example.

### Psychiatrist’s perspective

Physicians have always had sovereign power in medical diagnosis and treatment. This expertise will undoubtedly be strengthened by the use of AI-based systems for the time being in terms of optimization. Thus, it is already possible to provide more objectified and more complex diagnostics as well as personalized prognosis [17]—for example, referring to biomarkers (e.g. clinical, imaging, genetics), psycho-markers (e.g. personality traits, cognitive functioning), and social markers (type of social media use) in classifying certain mental disorders [17–19]. In the near future, psychiatrists will consciously and transparently shape their mediating role between the AI-generated expertise and the ethical decision-making process in the sense of patient autonomy.

### Patient’s perspective

In recent years, patients have matured into medical “lay experts” who use digital tools and the Internet in particular to acquire knowledge and derive actions or treatments. For example, AI-powered apps that are easily accessible to smartphone users expand patient empowerment in this regard and shape trust by making physicians’ actions verifiable [18]. How the free will to decide can be guaranteed, however, remains a central topic of the situational as well as the developing doctor–patient relationship. Not only physicians and patients grow and learn, but also ML or even Deep Learning (DL) are trainable technologies and, like humans, must be continuously subjected to the learning process [20]. As a consequence, this can also improve the interaction and trust relationship with AI.

For psychiatry, various ethical challenges (Table 1) to which the doctor–patient relationship is subject arise or intensify not only in the areas of prevention, diagnosis/prognosis, and therapy, but also in the areas of education and research.

**Table 1:** AI involvement in psychiatry: a selection of ethical challenges

	Ethical challenges	Type of AI involvement
Prevention	Harm prevention/safety [21], efficacy [22], data security [23]	Suicide prevention (e.g. based on the electronic health records) [24]
Diagnosis prognosis	Transparence (“black box”), data privacy [4], patient autonomy [18, 19]	Use of complex/multidimensional biomarkers [22], use of diagnostic apps
Therapy	Lack of human contact, safety [19]	Chatbots/robots as “therapists” [24]
Education	Responsibility [23], lack of guidance and training [21]	Digital competence in the field of AI necessary
Research	Participants’ data security, informed consent, data privacy, safety [25]	Study participation, data collection (e.g. monitoring)

## Conclusions

AI systems are currently one of the most important emerging technologies. Digital technologies should be considered not only as tools, but also as an acquired part of their users’ identity (e.g. viewing the smartphone as a “mobile identity”, i.e. a close, identity-forming connection between a person and technology) [26]. Accordingly, the goal should be not only to get lost in transhumanistic optimization, but also to follow the path of transformation. As we are in the midst of this change, the question is no longer “whether” technology should be used, but “how” we can use it to meet goals of progress or improvement. The focus should therefore always be on weighing technological possibility against ethical necessity. The European Commission’s ethical guidelines provide initial, but not exhaustive guidance, and the four principles of biomedical ethics provide a more concrete patient-centered and medical-practice view for AI systems in psychiatry. However, when considering the benefits and risks of using AI systems, it should always be checked whether the technology also stands up to ethical evaluation. In this context, Jotterand/Bosco (2020) basically claims that technological solutions should only be applied in medicine if they incorporate the ethical imperative of humanity and thus fulfill three requirements: technology serves human purposes, respects personal identity, and promotes human interaction [27].

## Data Availability

Not applicable.

## Abbreviations

AI: Artificial intelligence
COVID-19: Coronavirus disease 2019
DL: Deep learning
ML: Machine learning
